# Diagnoses for Charles Darwin’s Illness: A Wealth of Inaccurate Differential Diagnoses

**DOI:** 10.7759/cureus.32065

**Published:** 2022-11-30

**Authors:** John Hayman, Josef Finsterer

**Affiliations:** 1 Clinical Pathology, The University of Melbourne, Melbourne, AUS; 2 Neurology, Krankenanstalt Rudolfstiftung, Vienna, AUT

**Keywords:** cyclic vomiting, psychogenic illness, anxiety, helicobacter pylori, charles darwin illness, panic disorder, chronic fatigue, mitochondrial disorder, post-traumatic stress disorder, neurasthenia

## Abstract

Charles Darwin suffered from a relapsing, debilitating illness for much of his adult life with numerous, differing symptoms. His occasional problems as a student, his seasickness throughout the voyage of the *HMS Beagle*, and his brief illnesses when ashore in South America and Australia were all early expressions of this illness. Diagnoses for Darwin’s illness are as numerous as his symptoms and are equally variable. Many diagnoses reflect the medical fashion of their time; psychological and psychogenic diagnoses once flourished. These diagnoses have recently been comprehensively reviewed in an uncritical and unbiased account.

Rather than a repeat review of diagnoses this paper aims to critique and make a critical appraisal of the diagnoses given. As stated, they are not all right. Some are not wrong but are simply incomplete.

Pathological mitochondrial DNA (mtDNA) mutations are the cause of a variety of childhood diseases and more recently have been recognized as the cause of some adult-onset conditions with a plethora of presenting symptoms. The diagnosis favored here is that Darwin suffered from such a disorder due in his case to a maternally inherited pathological mtDNA mutation. This proposal should be seen in the context of *self-certainty *and subject to similar critical appraisal.

Diagnosing Darwin may have a unique, correct solution, a solution that would benefit those who suffer from a similar disorder today and who, like Darwin, are misdiagnosed, misunderstood, and inappropriately treated.

## Introduction and background

I of course believe in truth of my own doctrine, I suspect that no belief is vivid until shared by others. … When I think of the many cases of men who have studied one subject for years & have persuaded themselves of the truth of the foolishest doctrines, I feel sometimes a little frightened, whether I may not be one of these monomaniacs.

Charles Darwin, letter to Dr. William Carpenter, November 1859.

Charles Darwin (1809-1882), the famous naturalist, suffered a chronic, debilitating, relapsing illness for most of his adult life with numerous, diverse symptoms. The nature of this illness has been the subject of speculation and controversy dating back to Darwin’s lifetime. In a recent paper, Buchanan commented: “*In the 140 years since Darwin’s death, numerous diagnoses have been proffered in letters, articles, and a handful of dedicated books. The sheer number and variety of these conjectures is bewildering. And given the self-certainty that has accompanied many verdicts it might even seem comical. … we could surely conclude that something is amiss here, that they cannot all be right. We may even wonder if they are all wrong*” [1]. Jared Goldstein summed this up succinctly: “*Take your pick, there’s something for everybody: hypochondriasis, refractive error, depression, arsenic poisoning, Oedipal complex, pigeon allergy, familial psychosis, chronic brucellosis, chronic anxiety, Chagas’ disease, and more*” [2]. The diagnosis that is favored here, that of a mitochondrial disorder due to a maternally inherited pathological mitochondrial DNA (mtDNA) mutation, should be seen against this background and subject to similar critical appraisal [3].

Darwin’s illness: a brief history

Although minor, some symptoms of Darwin’s illness were already present before he was 16. At medical school in Edinburgh (1825-1827), it is recorded that any unpleasant news from home would cause him to lose “*a great many breakfasts*” [4]. His elder brother Erasmus (Erasmus Alvey Darwin 1804-1881) told him that cleaning up after an autopsy “*would not suit his stomack*” [5], and he was unable to watch surgical procedures (procedures that were certainly barbaric) because of what must have been intense nausea [6]. These accounts by themselves may not be significant except that there were similar occurrences later in life, once when he was present at the birth of his first son William [7] and again after he watched his second son, George’s, teeth extracted under chloroform [8].

Medical studies were abandoned. Darwin had further symptoms when a student at Cambridge (1829-1831). He had eczema (atopic dermatitis) of the face and hands and at least two episodes of severe incapacitating lethargy [6]. Newly qualified from Cambridge, Darwin was selected as a naturalist and a “gentleman companion” to Robert Fitzroy, captain of the *HMS Beagle* for a three-year, extended to a five-year, voyage that was to take them around the world.

While in Plymouth waiting to sail Darwin experienced “*palpitations and pain around the heart*,” symptoms that he kept to himself [6]. Throughout the voyage, he suffered from seasickness, not normal seasickness but an illness that became worse as the voyage progressed [9]. He had experienced the same sickness in 1825 when crossing the Channel on a visit to Paris with his Uncle Jos (Josiah Wedgwood II 1769-1843) and his future wife, then his cousin, Emma Wedgwood (1808-1896) [10].

Darwin was also ill several times when ashore, an illness that seemed to occur when he experienced more than one type of stress, such as physical exertion in the heat. He also had headaches, severe headaches, causing him to return to the ship on one occasion [11]. In Valparaiso, Chile, he was confined to bed for several weeks with a more prolonged illness. This illness differed from the illness that plagued him for most of his adult life, most likely this was typhoid or a typhoid-like (paratyphoid) fever [12]. After the voyage, he again suffered headaches, both before his proposal and before his marriage to Emma, the youngest daughter of Josiah II [13].

After his marriage in January 1839, the illness progressed, with an increased frequency of episodic nausea, retching, vomiting, and flatulence [14]. The family moved from London to Downe, in Kent, in the hope that the cleaner country air would improve Charles’ health. Alas, it did not. Along with his previous symptoms, Darwin experienced what has been diagnosed as panic attacks, waking at night feeling “*terribly afraid*” [15]. Added to recurrent headaches and palpitations, he experienced abdominal pain, sweating, giddiness, trembling, faintness, and visual disturbances. At times his lethargy was profound so that he could “*only lie on a sofa and do nothing*” [16]. Later in life, he had episodes of memory loss, inability to speak, and partial paralysis. He also had dying sensations and episodes of hysterical crying [17]. A list of Darwin’s symptoms, together with an interpretation of them, has been previously published [18].

He died with an illness different from that which had afflicted him for most of his life, with symptoms of heart failure, most probably due to coronary artery disease [14].

Diagnoses for Darwin’s illness

Diagnoses for Darwin’s illness are even more numerous than his symptoms were and just as variable. Proposed diagnoses date back to Darwin’s lifetime and continue up until the present day. Many of these diagnoses were popular at the time of their proposal and reflect concepts of illnesses current at that time. These diagnoses have been reviewed by Colp in two books [14,19] and, more recently, in their historical context, by Buchanan [1]. An updated, personal (JH) collection of diagnoses in chronological order, almost certainly an incomplete list, is given in the next section together with a critical analysis.

## Review

Diagnoses for Darwin’s illness: an appraisal

A list of diagnoses, listed according to the year of their first proposal and including their considered status, is given in Table 1.

**Table 1:** A chronological list of diagnoses for Darwin’s illness. Diagnoses for Darwin’s illness with their year of proposal and considered status. Obsolete: diagnosis no longer recognized; reject: diagnosis considered to be incorrect; incomplete: diagnosis reflects some symptoms but not the full spectrum of the illness; preferred: preferred diagnosis in this paper.

Year	Diagnosis	Reference	Status
1849	Nervous dyspepsia/aggravated dyspepsia	[20]	Obsolete
1849	Suppressed gout	[21]	Obsolete
1850	Malingering (“shamming”)	[22]	Reject
1863	“Waterbrash”	[23]	Incomplete
1865	Sequel to seasickness, voyage HMS Beagle	[24]	Reject
1888	Sequel to illness, Valparaiso	[25]	Reject
1901	Neurasthenia	[26]	Obsolete
1903	Refractive error	[27]	Reject
1918	Anxiety neurosis	[28]	Incomplete
1920	Psychogenic – repressed hostility father	[29]	Reject
1920	Psychogenic – latent homosexuality	[29]	Reject
1927	Reactivation birth trauma	[30]	Reject
1929	Pyorrhea	[31]	Reject
1943	Psychoneurosis	[32]	Reject
1954	Psychoneurosis – repressed hostility toward father	[33]	Reject
1958	Brucellosis	[34]	Reject
1959	Depressive psychosis	[35]	Reject
1959	Chagas’ disease	[36]	Reject
1963	Malaria	[37]	Reject
1963	Diaphragmatic hernia	[38]	Reject
1965	Paroxysmal tachycardia	[39]	Incomplete
1965	Psychogenic – unresolved grief of mother’s death	[40]	Reject
1966	Narcolepsy, “diabetogenic *hyperinsulinism*”	[41]	Obsolete
1971	Arsenic poisoning	[42]	Reject
1971	Mercury poisoning	[43]	Reject
1974	Allergy, pigeons	[44]	Reject
1977	Psychogenic – repressed hostility wife	[45]	Reject
1977	Porphyria	[46]	Reject
1987	Pyroluria	[47]	Reject
1990	Chronic fatigue (myalgic encephalomyelitis)	[48]	Incomplete
1990	Allergy	[49]	Reject
1994	Adrenal insufficiency	[50]	Incomplete
1997	*Lupus erythematosus*	[51]	Reject
1997	Panic disorder	[15]	Incomplete
1997	Ménière’s disease	[52,53]	Incomplete
1998	Psychological – father-son bonding	[54]	Reject
2000	Atopic dermatitis	[55]	Incomplete
2002	Obsessive-compulsive disorder	[56,57]	Reject
2005	Lactose intolerance (“systemic”)	[58]	Reject
2005	Asperger’s syndrome	[59]	Reject
2007	Crohn’s disease	[60]	Reject
2009	Psychogenic – abhorrence to slavery	[61]	Reject
2009	Cyclic vomiting	[62]	Incomplete
2009	Helicobacter infection	[63]	Incomplete
2012	Irritable bowel syndrome	[64]	Incomplete
2013	Candida overload	[65]	Reject
2014	Mitochondrial disorder	[66]	Preferred
2016	Post-traumatic stress disorder	[67]	Reject
2018	Chronic borreliosis (Lyme disease)	[68]	Reject

The diagnoses, as listed in Table 1, for the purpose of this appraisal, may be divided into several overlapping groups (Table 2).

**Table 2:** Categorization of the diagnoses for Darwin’s Illness, arranged for discussion. Diagnoses for Darwin’s illness grouped according to category. For completeness, some diagnoses appear in more than one category but are only discussed once in the text.

Category	Proposed diagnosis
Diagnoses made by Darwin and by his doctors, colleagues, and contemporaries	Nervous dyspepsia, aggravated dyspepsia, suppressed gout, malingering (“shamming”), waterbrash, sequel
Conditions supposedly acquired during his voyage with the HMS Beagle	Sequel to seasickness, voyage with the HMS Beagle, a sequel to prolonged illness Valparaiso, brucellosis, Chagas’ disease, malaria
Psychogenic, psychological diagnoses	Neurasthenia, anxiety neurosis, psychoneurosis, depressive psychosis; psychogenic – repressed hostility toward father, latent homosexuality, abhorrence to slavery; unresolved grief for mother’s death, father-son bonding; panic disorder, obsessive-compulsive disorder, post-traumatic stress disorder; Asperger’s syndrome
Intestinal disease and disorders	Diaphragmatic hernia, lactose intolerance (“systemic”), Crohn’s disease, cyclic vomiting (CVS), Helicobacter infection, irritable bowel syndrome (IBS)
Infections	Pyorrhea, brucellosis, Chagas’ disease, malaria, Helicobacter infection, chronic borreliosis (Lyme disease)
Diagnoses, alternative medicine	Pyroluria, Candida overload
Miscellaneous	Refractive error, arsenic poisoning, porphyria, lupus erythematosus
Correct, but incomplete diagnoses	Supraventricular tachycardia, chronic fatigue, adrenal insufficiency, panic disorder, Ménière’s disease, atopic dermatitis, cyclic vomiting (CVS), Helicobacter infection
Preferred diagnosis	Maternally inherited adult-onset pathological mtDNA mutation, MELAS type

In considering these various proposals, diagnoses that have already been systematically examined and rejected will not be discussed in depth, but their critiques will be summarized and referenced.

Diagnoses Made by Darwin and His Doctors, Colleagues, and Contemporaries

**Figure 1:**
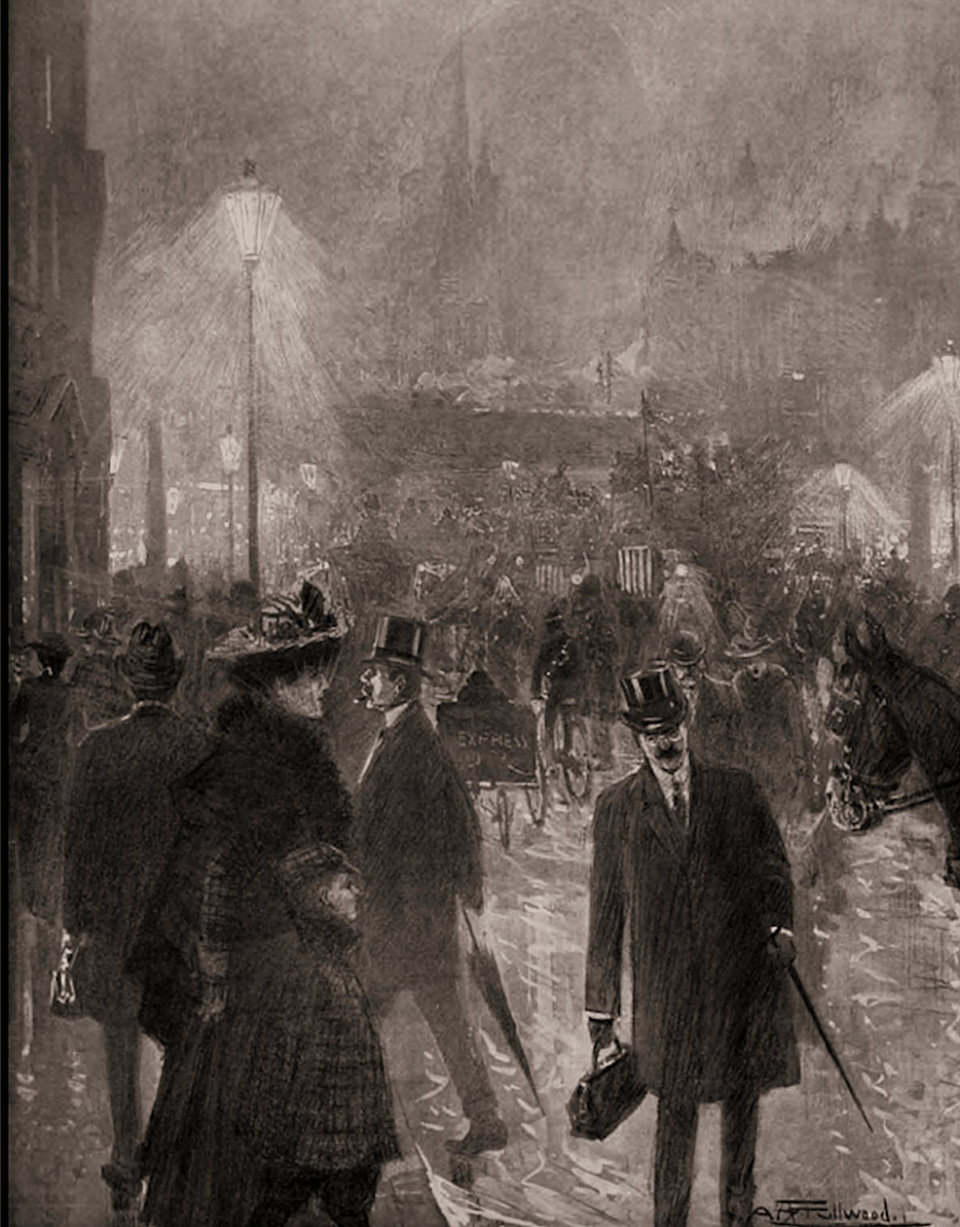
London smog, circa 1895. London as depicted by Albert Henry Fullwood (1863-1930) (Alamy stock photo).

In Darwin’s time, belief in sorcery and witchcraft had declined but there was still widespread conviction that disease was God’s punishment for sin [69]. Impure air (of which there was an abundance), and, more plausibly, dampness and cold were the essential causes of illness. Darwin moved to Downe to escape the putrid London environment, believing the less polluted country air would help his illness (Figure 1).

Diagnoses and treatments in Darwin’s time were empirical and directed toward symptoms rather than the often-unknown underlying disease. Gout was common; diagnosis of gout was even more common, modified with terms such as “suppressed” or “atypical” gout, “gout without joint involvement.” Hooker, in a letter to Darwin in 1862, wrote: “*Paget* (Sir James Paget 1814-1899) *told me that Eczema was a sort of nom de guerre for any skin complaint that had no other recognized name, - a sort of ‘suppressed gout’: I suppose, wh. means anything but Gout!*” Joseph Hooker, letter to Charles Darwin, July 1862.

Dyspepsia was another morbid condition, modified by terms such as “aggravated dyspepsia,” “atonic dyspepsia,” and “acid dyspepsia” [70]. Edward Lane, in his Reminiscences of Darwin, wrote: “*Mr. Darwin was at that time a great sufferer from dyspepsia of an aggravated character, brought on, as he always supposed, by the extreme sea-sickness he underwent in the course of his voyage round the world in H. M. S. Beagle* …” [20]. As these conditions are no longer recognized, these diagnoses may be safely discarded.

One term which is still with us is “waterbrash,” now “water brash,” a condition where there is increased salivation associated with the regurgitation of stomach contents into the esophagus and mouth. Darwin may well have had this symptom; he described himself as suffering from “retching” as well as actual vomiting, but this was one symptom only of a more complex disorder, not a diagnosis, as was proposed by Busk [23].

Diagnoses of Conditions Supposedly Acquired During His Voyage With the HMS Beagle

The most persistent of these diagnoses is that Darwin suffered from Chagas’ disease, a parasitic infection transmitted by various triatomine bugs. This diagnosis was first proposed by Adler in 1959 [36]. Woodruff made a comprehensive rebuttal of this diagnosis on epidemiological and clinical grounds, also pointing out that Darwin had symptoms before the voyage and those attacks of illness were brought on by “*emotionally charged situations*” rather than physical exertion as may have been expected with trypanosome myocarditis [71]. The diagnosis does not fit the medical picture. Despite this refutation, the diagnosis has remained a popular alternative to the various psychological proposals. Goldstein, in his appraisal of Darwin’s illness, favored this diagnosis, albeit with some reservations [2].

Other proposed voyage-acquired infections are brucellosis, malaria, and amoebiasis. Darwin may have experienced some of these infections when abroad but on clinical grounds alone they do not fit with the nature of his lifetime illness. These infections, if they had occurred, would not have lasted for years, producing symptoms over his entire remaining lifetime.

Apart from these infections, the seasickness endured throughout the voyage is thought by some, perhaps at one time by Darwin himself, as being the cause of his later illness [24]. Again, the prolonged illness experienced in Valparaiso, Chile, has been considered to have had lasting ill effects [25]. These, as well as the supposedly acquired infections, may be safely discarded if, for no other reason, Darwin had symptoms of illness before he set sail. The seasickness rather than being the cause was an intrinsic aspect of the illness.

Psychogenic/Psychological and Psychiatric Diagnoses

These diagnoses may be divided into two groups which may be psychogenic/psychological and recognized psychiatric disorders. The first group has been presented in depth by Buchanan and will not be reviewed individually here [1]. Psychogenic diagnoses once flourished, including numerous psychoanalytical diagnoses which have been proposed as the underlying cause for Darwin’s illness. Sir Hedley Atkins (1905-1983) was at one time President of the Royal College of Surgeons and he, with his wife Gladys, maintained Darwin’s home, Down House. He quoted several of these diagnoses and scathingly referred to them as a lot of psychoanalytical “clap-trap,” using that term to denote the *promulgation of a theory which, whether* *true or not, has not a vestige of scientific evidence to support it* [72]. Both he and his contemporary Sir George Pickering FRS (1904-1980), Professor of Medicine at Oxford, believed that Darwin had a primary psychological illness, as did Woodruff in his rebuttal of the Chagas hypothesis [71]. In fairness to these three outstanding clinicians, they would not have known of mitochondrial disorders, the preferred diagnosis here, as the adult forms of these conditions were not recognized in their time.

The latest edition to this genre is the book by Desmond and Moore, proposing that Darwin’s illness arose from his abhorrence of slavery [61]. This thesis was systematically refuted by Esterson, who wrote “*Desmond and Moore have a propensity to give truncated quotations within paragraphs that frequently enable them to create an impression of providing substantive evidence in support of their central thesis*” [73].

In contrast to these psychological proposals, Goldstein was assured that Darwin did not have such an illness. He wrote: “*He* (Darwin)* comes through to me now as a steady, serene, and cheerful person. It is this Darwin that some claim inflicted his own illness upon himself through some psychological flaw. The more I read of his letters, the better I feel that I know him, and the more I become convinced that his chronic illness was organic in origin*” (“organic,” in this context, implies organ specific). The authors agree with Goldstein in the belief that Darwin did not have a primary psychological illness.

Although Darwin did not have a primary psychological illness, he did have psychological symptoms. He had anxiety about how his book would be received; he had anxiety that his children may have inherited his illness; and he had anxiety as part of his illness - he was anxious to avoid attacks of sickness and he had nervousness when Emma left him. Darwin also had attacks of fear, waking in the night “feeling terribly afraid” and other symptoms of a panic attack. He had episodes of extreme lethargy and hysterical weeping. The attacks of fear may have been the sequel to neuroendocrine dysfunction, relating to the hormone cholecystokinin [18]. The episodes of weeping he experienced represent dacrystic seizures, a form of epilepsy rather than a psychiatric symptom [74]. He certainly had periods of dejection because of his illness and may at times have had actual depression. Lethargy and depression are common symptoms in patients with known mitochondrial disorders [75].

Furthermore, psychological as well as physical stressors would precipitate episodes of illness. Darwin wrote: “… *anything which flurries me completely knocks me up afterwards and brings on a b⁠ad⁠⁠ palpitation of the heart*” [76]. In contrast, Darwin’s health improved when he was resident in Dr. Gully’s establishment in Malvern and psychological stresses were absent. Writing to his friend and mentor Henslow (Rev John Stevens Henslow 1796-1861) Darwin recorded: “*One most singular effect of the treatment is, that it induces in most people, and eminently in my case, the most complete stagnation of mind: I have ceased to think even of Barnacles*!” [77].

Primary psychiatric, as well as psychological or psychogenic disorders, have also been proposed, in particular, depressive illness [35]. George Pickering, who himself suffered from depressive illness, in his book Creative Malady, considered that Darwin at times suffered from dejection rather than true depressive illness [12]. Darwin’s prodigious work output and abundant correspondence were quite unlike those of a person with such an illness.

Gastrointestinal Diagnoses

These include reflux disease, diaphragmatic hernia, lactose intolerance, Crohn’s disease, gastritis with *Helicobacter *infection, and gastric and irritable bowel syndrome (IBS) [38,58,60,63,64]. These diagnoses might account for some of Darwin’s symptoms but not for the whole spectrum of his multisystem illness. Furthermore, there are some specific points that might cause doubt about these diagnoses.

*Helicobacter *infection was proposed by Barry Marshall who shared the Nobel Prize in physiology or medicine in 2005 for the discovery of the bacterium *Helicobacter pylori* and its role in gastritis and peptic ulcer disease [78]. Marshal proposed *Helicobacter* infection as the cause of Darwin’s illness. As he himself wrote: Like almost everyone in Victorian England, and the rest of the world, Charles Darwin most likely carried *Helicobacter*. Of people with *Helicobacter*, 10% have a chronic peptic ulcer in their lifetime and maybe an equal percentage have chronic dyspepsia. Darwin may well have had a *Helicobacter *infection but although this might have caused some symptoms it would not account for his entire symptomatology, particularly his neurological symptoms. Darwin’s attacks of illness were brought on by different stresses, including infection. A flare of *Helicobacter *gastritis could well have brought on further symptoms.

More specifically, Darwin received many different substances in an attempt to alleviate his illness, including bismuth. Bismuth remains one of the most effective treatments for *Helicobacter *infection [79]. It may have been of some initial benefit but was not curative. To his sister Susan he wrote: I have taken my Bismuth regularly, I think it has not done me quite so much good, as before … [80]. Later, his wife Emma records in her diary (August 17, 1864) “began bismuth &c” [81]. The use with some apparent benefit from bismuth supports the contention that Darwin did have *Helicobacter *infection and the probable associated gastritis.

Crohn’s disease, a condition that more commonly affects the terminal ileum, was confidentially proposed as a cause of Darwin’s illness in 2006 [60]. This diagnosis was comprehensively dismissed on clinical grounds by Sheehan, Meller, and Thurber who pointed out that Darwin’s main gastrointestinal symptom was vomiting, an unusual feature with Crohn’s disease, and that his notes for his physician “*hardly sounds like Crohn’s* *disease*” [82]. The diagnosis was later resurrected on a result of DNA analysis of some hairs from Darwin’s beard, showing he had DNA that may be associated with Crohn’s disease, but this evidence also was far from convincing [83]. This diagnosis alone does not account for Darwin’s severe headaches, visual disturbances, muscle fasciculation, attacks of fear, episodes of memory loss, hysterical weeping, palpitations, and heat and cold intolerance.

IBS is another proposal that might account for some symptoms [64], but IBS symptoms are predominantly lower bowel while Darwin’s symptoms were more stomach and upper bowel in type [84]. IBS is associated with constipation or diarrhea, sometimes alternating. In his notes for his physician (1865), Darwin declares “*Evacuation regular & good*” [17], a statement that would seem incompatible with IBS as a diagnosis.

Lactose intolerance is another persistent diagnosis although there appears to be little evidence to support it. Darwin’s seasickness certainly improved when his diet was just raisins, a change recommended by his father [85]. This seasickness was an inherent part of the illness, not a “red herring,” as had been proposed [58]. Much later, Darwin also improved when enduring hydrotherapy in Dr. Gully’s establishment in Malvern where the therapy included a diet from which “*sugar, butter, spices tea bacon or anything good*” was excluded. Darwin however was allowed “*a little milk to sop the stale toast in*” [86].

Different forms of stress would bring on illness rather than any particular food ingredient. The benefit from Malvern appeared largely due to “*the most complete stagnation of mind*” that Darwin experienced in that institution [77]. When Darwin continued the water treatment at his home in Downe stresses would have again been present; there the treatment was apparently less successful and was discontinued.

There are numerous accounts of stress bringing on illness in Darwin’s correspondence: “… *anything which flurries me completely knocks me up afterwards and brings on a b⁠ad⁠⁠ palpitation of the heart*” [76], and after giving a paper on the sex lives of orchids to the Linnean Society: “*I by no means thought that I produced a ‘tremendous effect’ on Linn. Soc; but by Jove the Linn. Soc. produced a tremendous effect on me for I vomited all night & could not get out of bed till late next evening, so that I just crawled home. - I fear I must give up trying to read any paper or speak. It is a horrid bore I can do nothing like other people*” [87]. Minor stresses, even congenial events would bring on illness. After a dinner party in their home, Emma reported: “*Charles was dreadfully exhausted when it was over, and is only as well as can be expected to-day. ... He is rather ashamed of himself for finding his dear friends such a burden*” [88].

Darwin, in the course of his long illness, tried many different treatments, including dietary regimes. He would surely have noticed if avoiding milk or dairy products improved his health. His health did improve in his later years, but this was because he learned to avoid stressful situations and, apart from family and close friends, had few visitors. Emma’s diary contained recipes for skim milk pudding, Miller’s pudding, burnt cream, Crème a la Victoire, stone cream, almond puddings, Lady Skymastons pudding, etc., all containing generous quantities of milk or cream [89]. Darwin enjoyed his meals and there is no evidence that any of his wife’s recipes were curtailed in his older age.

Infections

Various infections have been proposed as the cause of Darwin’s illness. Those supposedly acquired during the voyage of the *HMS Beagle* (Chagas’ disease, brucellosis, malaria, amoebiasis, dengue, trypanosomiasis, onchocerciasis, etc.) are discounted based on Darwin having symptoms of illness before this voyage, and, furthermore, these diagnoses do not fit with the clinical features of the illness [71]. Darwin’s son, Leonard, offered his opinion that his father was constantly ill from the systemic effects of pyorrhea (periodontal disease) [90]. The concept of focal infection, particularly dental sepsis, as a cause of systemic illness was en vogue in the early 20th century but has largely been discarded [91,92].

More recently, Lyme disease (chronic borreliosis) has been proposed as the cause of Darwin’s suffering [68]. Lyme disease is a bacterial infection caused by *Borrelia burgdorferi* or related organisms and transmitted to humans from animal reservoirs through tick bites [93]. The infection typically causes dermatologic, musculoskeletal, neurologic, and cardiac symptoms. While antibiotic therapy resolves these symptoms for most infected patients, some are left with persistent subjective problems, such as pain, fatigue, or brain fog, which may fall within the proposed entity post-treatment Lyme disease syndrome [94]. The condition is frequently misdiagnosed in patients with chronic ill health with subjective symptoms and the term “chronic Lyme disease” has been adopted by alternative health practitioners.

“Chronic Lyme disease” was proposed on the basis that the young Darwin spent time in the woods when hunting and could have been bitten by a tick. There is no record of this happening and no record of acute illness or the skin rash that may occur with the acute infection.

Alternative Medicine

Alternative therapies often flourish where orthodox medicine is unable to provide effective treatment, particularly in the field of advanced or disseminated cancer. Pyroluria, the proposed background for a wide range of disorders, has been proposed as the cause of Darwin’s illness [47]. Pyroluria is one such hypothetical “orthomolecular” disorder, with excessive pyrroles in the body supposedly the result of defective hemoglobin synthesis [95]. The scientific medical literature has no evidence that such a condition exists. Signs and symptoms of this ailment are such that all of us would have at least one symptom of the condition. For example: “did you get a ‘stitch’ in your side when you ran as a child?” or “are you easily tired?.” However, there are a few more specific signs. One is the presence of “bushy” eyebrows, which Darwin certainly had, and an inability to remember dreams. Darwin, on many of the diagnostic criteria proposed, would certainly have had pyroluria. Symptoms of pyroluria are eczema, tingling sensations, cold hands and feet, anxiety, socially withdrawn and dependent fairly strongly on one person (with Darwin, this would be his wife Emma), and a member of his immediate or extended family who had committed suicide (in Darwin’s case, possibly his maternal uncle, Tom). However, Darwin could remember, and several times actually recorded his dreams. For example, on *October 30,* 1838 - “*Dreamt somebody gave me a book in French I read the first page & pronounced each word distinctly, woke instantly but could not gather general sense of this page*” [96].

Another proposed diagnosis is systemic candidiasis or “candida overload” [65]. Systemic candidiasis is also frequently diagnosed as the intrinsic cause of a variety of complaints (even in the absence of any sign of yeast overgrowth). These patients too are said to have headaches, lethargy, reduced concentration, indigestion, heartburn, “food allergies,” skin rashes, joint soreness, and mood swings. As is confidently stated, millions of us suffer from this disorder and remain undiagnosed [97].

Systemic candidiasis may occur in immunologically compromised individuals but there is no evidence that Darwin ever had sustained immunological deficiency. Furthermore, so-called polysystemic candidiasis remains an unproven entity [98].

Miscellaneous Diagnoses

Several proposed diagnoses may be contained under this heading. Two, unrelated conditions, porphyria and lupus erythematosus (systemic lupus erythematosus, SLE) produce changes in the urine. In the course of his long illness, Darwin consulted many doctors, including Headland (Frederick William Headland 1830-1875) and Bence Jones (Henry Bence Jones FRS 1813-1873), both noted for their studies of urine [99,100]. Darwin himself noted that “Urine scanty (because do not drink) often much pinkish sediment when cold …” [17]. (The sediment may have been crystalline phosphate compounds, which precipitate in cold urine.) Porphyria produces a purplish color change in the urine; SLE is associated with protein (proteinuria). Darwin’s urine was certainly examined and these changes, if present, would have been noted.

Although the prognosis of SLE has improved today, in Darwin’s time, the condition would have had a poor outlook [101]. He would not have lived to the age of 73 with this diagnosis. Furthermore, the suggestion that Darwin’s recurrent boils were in fact skin nodules that may occur in SLE is unlikely as SLE nodules are characteristically painless.

Refractive eye error was one of the earlier but less likely diagnoses proposed for Darwin’s illness [27]. Dr. Milbry Gould (George Milbry Gould 1848-1922) specialized in treating such refractive errors. In a book published in 1903, he diagnosed Darwin (along with other notables) as suffering from a refractive error and subsequent eyestrain [27]. Gould ascribed Darwin’s seasickness to eyestrain from using a microscope on the long voyage, conveniently omitting the fact that Darwin had vomited as soon as they sailed, and on the earlier attempts to put to sea when Darwin’s instruments were packed in drawers in his tiny cabin. Edward Lane, in his Recollection of Darwin, recorded: “*systematic exercise was one of the great means relied on for the cure of chronic diseases, and it was in the course of the long country rambles thus necessitated, that Darwin was seen at his very best. He was then literally *‘*all eyes.*’* Nothing escaped him. No object in nature, whether Flower, or Bird, or Insect of any kind, could avoid his loving recognition*” [20]. Hardly the picture of a man who needed glasses.

The contention that Darwin suffered from chronic arsenic poisoning has been the subject of an entire book, albeit a small book [42]. We know that arsenic was a common remedy in the early 19th century and was prescribed for a variety of complaints including headaches, “brow ague,” probably a form of malaria, and numerous skin conditions [102]. Arsenic was used also as a tonic, giving “firmness and vigor to the constitution.” An early sign of toxicity was the flushing of the cheeks, making the patient appear healthier. Darwin certainly took arsenic at one time for his skin complaints, as evidenced by his companion at Cambridge, John Herbert (John Maurice Herbert 1808-1882), and we know that he asked his father about taking arsenic for his hands when waiting to sail from Plymouth [102,103]. His father advised him against arsenic and his father’s advice was followed [104]. Foster, in a review of the book, was critical of the evidence presented and was unable to confirm some details. He concluded that he was unconvinced by the “ingenious hypothesis” [105]. Besides this, Darwin’s eczema, for which he took arsenic at least for a short period, was part of his overall illness. Symptoms of his illness preceded this one treatment so logically arsenic could not have been the cause.

Incomplete Diagnoses

The diagnoses that have been considered are thought to have been in error; several diagnoses, however, accurately reflect some of Darwin’s symptoms. As such, they cannot be regarded as incorrect but simply as incomplete, they do not cover the full spectrum of the illness. Darwin’s eczema has been diagnosed as atopic dermatitis [55], and with it he suffered recurrent boils, a frequent association [106]. He also experienced infected scratches when in the tropics. He suffered from panic attacks and other symptoms that may be considered part of a panic disorder [15]. His palpitations were the result of supraventricular tachycardia (paroxysmal tachycardia) [39]. He had symptoms of migraine with headaches and visual disturbances, and he had vestibular disturbances with dizziness [52.53]. Darwin had symptoms of chronic fatigue and fibromyalgia [48], IBS [64], and CVS [62]. Patients diagnosed today with CVS often experience motion sickness, as did Darwin with his seasickness, and have attacks brought on by stress, including pleasurable events (“positive stress”), again characteristic of Darwin’s illness [107].

**Figure 2:**
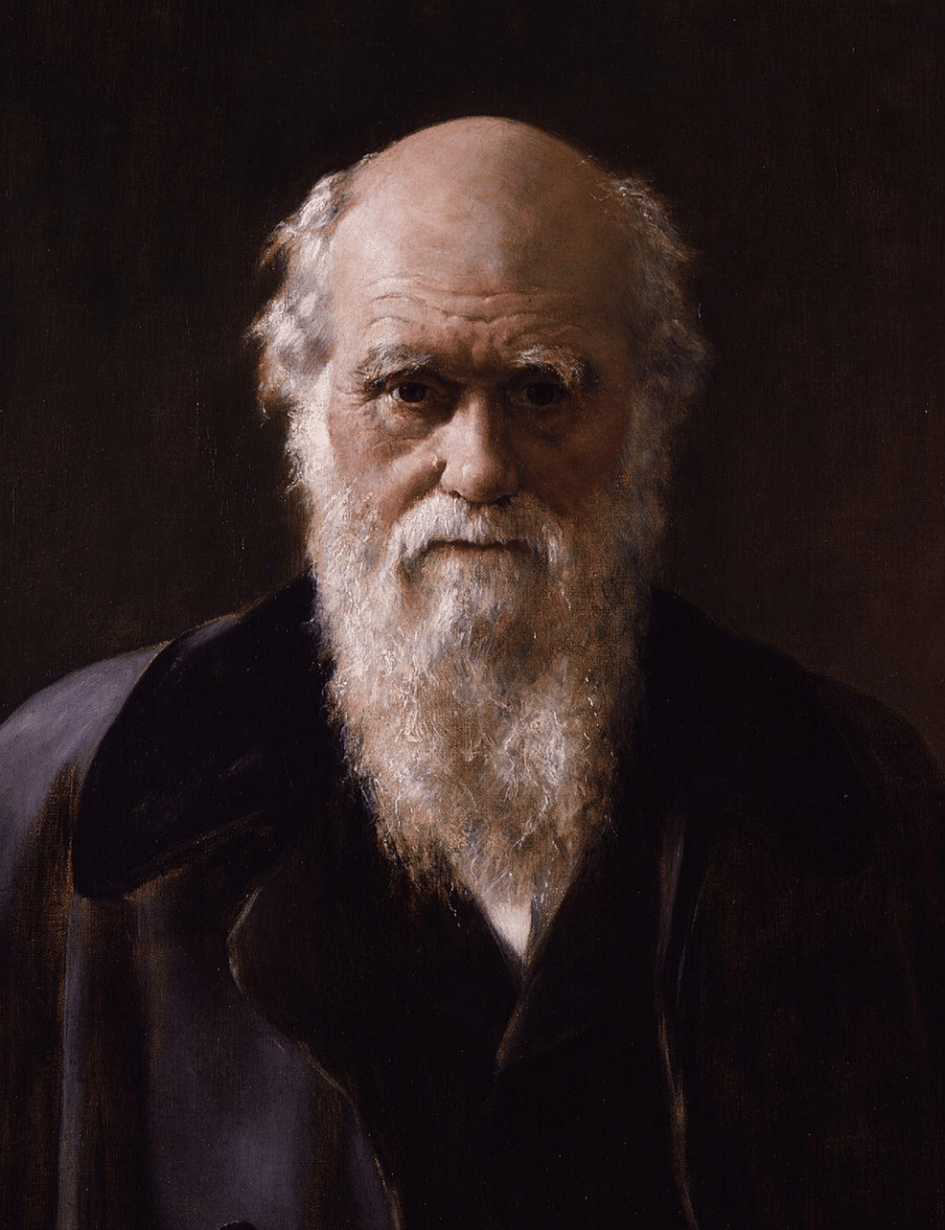
Charles Darwin, from the painting by John Collier, painted in 1881 (Wikimedia Commons). This portrait, taken from the painting, shows Darwin’s well-developed skin tan, an attribute which made him appear healthy, hiding his frequent sufferings.

Darwin had one feature of adrenal insufficiency (Addison’s disease) [50]. He developed skin pigmentation, a skin tan, which he described as “ruddy,” making him appear more healthy than he was. It is the same type of pigmentation as is seen in adrenal insufficiency, due to increased levels of adrenocorticotropic hormone/melanocyte-stimulating hormone secretion. In Darwin’s case, this increased secretion was the consequence of salt and fluid loss from repeated vomiting, not due to primary adrenal gland disease. This pigmentation is seen in the famous painting by John Collier, painted in 1881 (John Maler Collier 1854-1934) (Figure 2).

All of these ailments have been proposed as the diagnosis for Darwin; all account for some of Darwin’s symptoms but none alone account for the full range of his complaints [18]. It is, of course, possible that Darwin had several concurrent lifetime illnesses, but this would seem improbable; one diagnosis to account for all is preferable.

Preferred Diagnosis

The preferred diagnosis for Darwin’s illness is that of a maternally inherited pathological mtDNA mutation of the MELAS type [3]. A less precise but more memorable diagnosis would be “*Darwin’s Illness: Mitochondrial, not Hypochondrial*.” This diagnosis accounts for all of Darwin’s symptoms or complications of these primary symptoms [18].

The diagnosis also explains the strange symptoms of Darwin’s elder brother Erasmus (Erasmus Alvey Darwin 1804-1881), who suffered lethargy and abdominal pains, and of their female siblings, who developed chronic illnesses. It explains the illness of their mother Susannah (Susannah Darwin, née Wedgwood 1765-1817) who famously was “never quite well & never very ill” [108]. The diagnosis accounts for the illness of her younger brother Tom Wedgwood (1771-1805) who suffered severe headaches, abdominal pains, and seasickness similar to that of his nephew much later [109]. The index case is Mary Anne (Mary Anne Wedgwood 1778-1786), the youngest sibling of that generation who died at the age of eight with typical MELAS [110], one of the first described mtDNA disorders [111]. Other members of that generation developed their illnesses in later life; illnesses also linked to mitochondrial dysfunction [66]. Their mother, Sarah, Charles Darwin’s maternal grandmother, in turn, had a chronic illness consistent with a mitochondrial disorder. This familial, maternal pattern of illness strongly supports the proposal of an inherited mitochondrial disorder.

The preferred diagnosis does not explain the illnesses of Charles Darwin’s children who were indeed a sickly lot. Their mitochondria, as do all mitochondria in humans, came from their mother. They did not inherit their father’s mitochondria, nor could they have inherited from him any mitochondrial disorder. Darwin’s children’s illnesses were essentially different from that of their father and different from each other [112]. The seven children who survived infancy and childhood grew into essentially healthy adults whereas their father was a healthy child becoming unwell in later life.

Mitochondria and Mitochondrial Disease

Mitochondria produce energy for the cell, energy in the form of ATP. Cells vary in the number of mitochondria they contain, from several hundred to several thousand, depending on the cell’s energy requirements [113]. Unlike other cell organelles, mitochondria contain their own DNA, mtDNA. MtDNA mutates more commonly than nDNA; cells frequently contain both “wild” (normally functioning) DNA and mutant mtDNA, a condition referred to as heteroplasmy. When a cell divides, mitochondria flow randomly to daughter cells so that these may vary in their levels of heteroplasmy.

Mitochondria are maternally inherited in humans; the few mitochondria present in the sperm do not survive in the fertilized ovum. Mature ova contain thousands of mitochondria. Due to a reduction then, a proliferation during maturation ova from the same mother and the same ovary varies considerably in heteroplasmic levels.

Unlike genetic disease due to nDNA mutation, disease due to pathogenic mtDNA mutation does not depend on the nature of the mutation but rather on the level of heteroplasmy and the distribution of the mutant DNA in the tissues of the body [113]. Thus, different mutations may result in similar symptoms and the same mutation may result in very different symptoms. Due to the heteroplasmy variations in the original ovum, pregnancies in the same mother may result in spontaneous abortion, early fatal childhood disease, progeny developing disease in adult life, or in apparently healthy individuals.

With a mitochondrial disorder, it is the function of cells with high energy requirements that is affected. These include cerebral and autonomic nerve cells, cells of the enteric nervous system, cerebral endothelial cells, cardiac conduction cells, cardiomyocytes, retinal ganglion cells, and neuroendocrine cells [18]. Dysfunction of these cells produces symptoms in different body systems, resulting in a multisystem disorder.

## Conclusions

Diagnoses for Darwin’s illness show how much medicine has changed since his lifetime. A maternally inherited pathological mtDNA mutation provides a comprehensive explanation for Charles Darwin’s multisystem disorder. This diagnosis also explains the chronic illnesses that afflicted Darwin’s Wedgwood forebears, an aspect not considered by other propositions.

In making a preferred diagnosis we are conscious of previous diagnoses put forward with absolute certainty. Just as this paper is critical of other proposals, we welcome critical appraisal of this diagnosis. This diagnosis, directly or indirectly, accounts for all of Darwin’s symptoms, not a selected favorable proportion.

In making this diagnosis we hope that it is of help to those who suffer from a similar disorder today. Like Darwin, they suffer from misdiagnosis, lack of understanding, and endure inappropriate treatment. Adult-onset mitochondrial disorder remains a poorly recognized condition. Although curative treatment is not available, correct diagnosis and empathy allow for alleviation of distress. In the future, new techniques with gene editing may provide effective treatment and nuclear transfer in the ovum offers a means of prevention of mitochondrial disorders.

The authors would like to think that Darwin would accept this diagnosis, and even be pleased with it. He would never have accepted that his “illness was all in his mind.” Of course, he never knew of mitochondria or of their fascinating evolutionary history, but he would have been delighted to learn of this and intrigued that his illness was related to an event that occurred more than one billion years ago.

I think I shall convert 4 or 5 really good judges and that will content me, as I feel sure that they are too good judges to be deceived, and in course of years others will come round. If 4 or 5 good judges are not converted, then I may be a monomaniac.

Charles Darwin, letter to his sister Caroline Wedgwood, November 1859.

Requiescat in pace Carolus Darwin.
